# The antidiabetic and hepatoprotective effects of myricitrin on aged mice with D-galactose 

**Published:** 2020

**Authors:** Mina Omidi, Akram Ahangarpour, Layasadat Khorsandi, Fatemeh Ramezani- AliAkbari

**Affiliations:** 1 *Student Research Committee, Ahvaz Jundishapur University of Medical Science, Ahvaz, Iran*; 2 *Department of Physiology, Health Research Institute, Diabetes Research Center, School of Medicine, Ahvaz Jundishapur University of Medical Sciences, Ahvaz, Iran *; 3 *Department of Anatomical Sciences, School of Medicine, Cell & Molecular Research Center, Ahvaz Jundishapur University of Medical Sciences, Ahvaz, Iran*

**Keywords:** Myricitrin, Liver, Pancreas, D-galactose, Aging

## Abstract

**Aim:**

The present study aims to evaluate the effects of antidiabetic and hepatoprotective of myricitrin in the aged mice induced by D-galactose (D-gal).

**Background:**

Aging occurs during a person’s life; there has been no way to stop the aging process, but antioxidant and changing lifestyles can delay it.

**Methods:**

In this experimental study, 72 female adult mice (weighing30–35g) were randomly divided into six groups: 1: control, 2: D-gal at 500mg/kg/d, 3-5: D-gal+ Myricitrin at 5, 10 and 20mg/kg/d 6: D-gal+ Vitamin E at 100mg/kg/d. Aging induced by D-gal for 45 days via intraperitoneal. Myricitrin and Vitamin E administrated orally by gavage for the last 28 days. The blood glucose, insulin level, β-cell function, insulin resistance, hepatic enzymes, lipid profile, and histology of the liver, and pancreas were evaluated.

**Results:**

D-gal injection increased the glucose (p<0.001) and insulin levels (p<0.01) compared to control group. Myricitrin (p<0.01) and Vitamin E (p<0.001) increased insulin and decreased blood glucose levels compared to D-gal group. Myricitrin had a similar impact on insulin levels to vitamin E. Insulin resistance induced in the D-gal group (p<0.001). Myricitrin reduced insulin resistance and increased β-cell function (p<0.01) compared to D-gal group. D-gal elevated (p<0.01) cholesterol, LDL and triglyceride level, myricitrin (p<0.001), and Vitamin E (p<0.05) were reduced.

**Conclusion:**

D-gal-induced aging causes the accumulation of RBCs, inflammation in the liver, and changes in the number and diameter of Langerhans islets in the pancreas. Myricitrin improved these D-gal effects. Myricitrin had the anti-diabetic and hepatoprotective effects on the aged mice induced by D-galactose.

## Introduction

 Aging is a biological process associated to dysfunction in various organs of the body during a person’s life which varies from person to person. It is caused by pathological and psychological changes in individuals (1). There is no way to stop aging process, but it is delayed by antioxidant and changing lifestyles (2). Aging is among the most significant causes of chronic diseases, including diabetes, Alzheimer, and cardiovascular disorders. It demonstrated that aging caused pancreatic beta-cell dysfunction. Aging leads to insulin resistance results from increased free radical production and oxidative stress (3). A previous study showed that injection to D-galactose (D-gal) in the rats and mice could induce a model of aging and elevate oxidative stress by free radical generation (4). Furthermore, it has been reported that D-gal was caused by diabetes (5). Gradual impairment in the liver and pancreas' function and structure occurs in the aging process (6, 7). Today, special attention has been paid to using herbs and antioxidants to control and improve diabetes, which has been a concern in this field of much research worldwide (8).Oxidative stress is an imbalance between antioxidant defense and free radical production which causes excessive release of free radicals and damage (9). Myricitrin is an herbal flavonoid and found in various species of plants including Myrica Cerifera. This strong antioxidant property is due to the presence of compounds such as myricetin and polyhydroxy (10, 11). A previous study indicated that myricitrin caused various cell protection against damage, tumor growth inhibition, and nitric oxide production (12). Myricitrin administration led to decreasing oxidative stress induced by CCl-4 in the hepatocyte through increasing glutathione activity, hydrogen peroxide, and nitric oxide radical reduction (13). Thus, the present research designed to assess the effects of antidiabetic and hepatoprotective of myricitrin on aged mice induced by D-gal. 

## Methods

**Drugs**

Myricitrin was purchased from AvaChem San Antonio, U.S.A (purity 98%). D-gal, (Merck, Germany), progesterone (Iran hormone, Iran), Estradiol valerate (Aburaihan, Iran), and vitamin E (Osve, Iran) bought from multiple companies. 

**Animals and experimental design**

In this experimental study, 72 female NMRI (Naval Medical Research Institute) mice with weighing 30-35 g (12 weeks) purchased from the animal facility Ahvaz Jundishapur University of Medical Sciences (AJUMS). Animals were treated to methods agreed on by the animal care of AJUMS with the ethical number (IR.AJUMS.REC.1397.003). All animals had free access to tap water and food. They were stored at 22 ± 2˚C with a 12/12-hour light-dark cycle. The mice were accidentally divided into six groups (12 per each group); 1: control, 2: D-gal at 500mg/kg/d, 3: D-gal+ Myricitrin at 5 mg/kg/d, 4: D-gal+ Myricitrin at 10 mg/kg/d, 5: D-gal+ Myricitrin at 20 mg/kg/d, 6: D-gal+ Vitamin E at 100mg/kg/d. 

**Estrous cycle evaluation**

Steroid hormones can affect plasma insulin alterations and glucose metabolism in female rats by estrus cycle. Thus, one experimental way applied to produce the same estrus cycles in female mice.

Estradiol valerate at 100 mg and after 42 hours, progesterone at 50 mg intramuscularly (each hormone dissolved in 0.2 ml olive oil solution) were injected into the mice. Passing six hours, after the injection of progesterone, the vaginal smear was prepared. Then smears were dyed with methylene blue on the slides and observed using a light microscope) 5).

**Experimental measurement**

Finally, after the last injection of drugs (D-galactose, Myricitrin, Vitamin E), the animals anesthetized with ketamine-xylazine. The blood samples were taken from the heart and then centrifuge at 3000 rpm for 20 min, plasma samples stored at -80°C until hormonal assessment. Glucose and lipid profiles measured by biochemical assay kits (Iran, Pars Azmoon), and insulin levels were determined utilizing ELISA assay kits (USA, Monobind) (The sensitivity of hormone was detected by detection limit of 0.182 μIU/ml in each test tube (. Also, Homeostatic Model Assessment for Insulin Resistance (HOMA-IR), homeostasis model assessment of β-cell function (HOMA-β), were calculated by following formula (14, 15):

HOMA-IR: Fasting blood glucose (mg/dl) × insulin (μIU/mL)/ 405

HOMA-β: 100 × insulin (μIU/mL)/ (FBS (mMol/L) – 3.5) 

**Lipid profiles and hepatic enzymes measurement**

The plasma level of total cholesterol (TC), low-density lipoprotein (LDL), triglyceride (TG), high-density lipoprotein (HDL), plasma glutamic pyruvic transaminase (SGPT), glutamic oxaloacetic transaminase (SGOT) and level of alkaline phosphatase (ALP) were measured using commercial kits (Iran, Pars Azmoon). Very low-density lipoprotein (VLDL) level assessed using the Norbert formula (equals one fifth of TG content) (16).

**Histological assessment**

After blood collection, the liver and pancreas tissues were immediately removed and fixed in 10% formalin solution. Then, dehydrated in graded alcohol concentrations and embedded in paraffin. The considered sections by 4-6 µm were prepared and stained with hematoxylin and eosin (H&E). The slides examined to assess histological changes of the liver tissue, such as the accumulation of RBCs and the infiltration of inflammatory cells. The histological features were graded into 4 categories: normal (0), weak (1), moderate (2) or intense (3), and the averages were considered. For each slide, the mean of 6 fields was computed, and the slides were read in a “blind” fashion (17). 

In the pancreas tissue, the diameter of islets of Langerhans assessed using Motic Images plus 2.0 image analysis software (18). Islet numbers were counted using a quantitative-stereological method, as previously described (19).

**Statistical assessment**

The data were presented as mean± SEM and analyzed using SPSS software. One-way ANOVA and post hoc Tukey test used to assess the differences among groups. P value<0.05 was statistically significant. 

## Results

*The effect of myricitrin and vitamin E on fasting blood glucose, insulin level, and insulin-related biomarkers*

**Figure 1:**
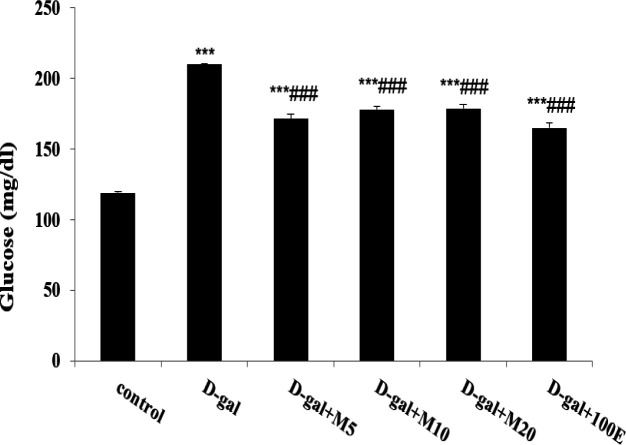
The effect of myricitrin and vitamin E on fasting blood glucose levels (mg/dl). Data expressed as mean ± SEM. ***p<0.001 as compared to the control group; ###p<0.001 as compared to the D-gal group, n=12 (One-way ANOVA and Tukey’ test).

**Figure 2:**
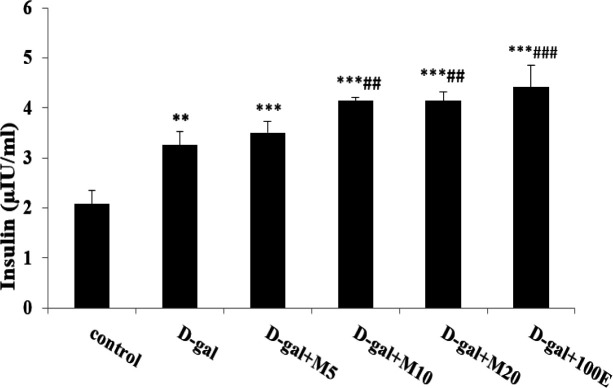
The effect of myricitrin and vitamin E on insulin level (µIU/ml). Data expressed as mean ± SEM. **p<0.01 and, ***p<0.001 as compared to control group; ##p<0.01 and, ###p<0.001 as compared to D-gal group, n=12 (One way ANOVA and Tukey’ test).

**Figure 3:**
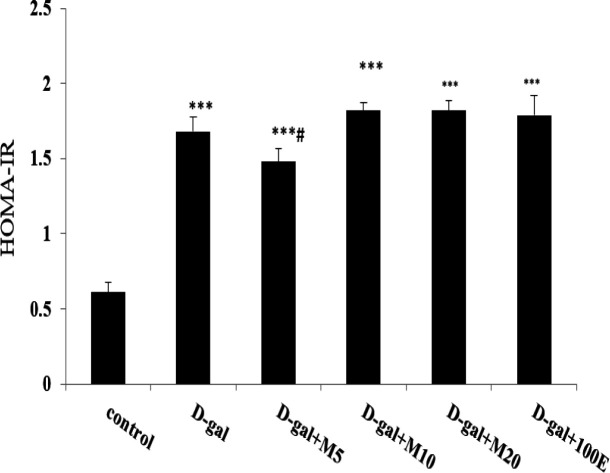
The effect of myricitrin and vitamin E on HOMA-IR. Data expressed as mean ± SEM. ***p<0.001 compared to the control group; #p<0.05 compared to D-gal group, n=12 (One way ANOVA and Tukey’ test)

**Figure 4:**
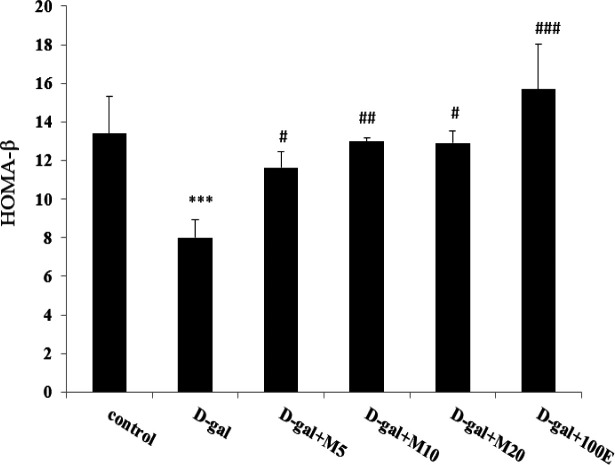
The effect of myricitrin and vitamin E on HOMA-β. Data expressed as mean ± SEM. ***p<0.001 as compared to the control group; #p<0.05 and, ###p<0.001 as compared to the D-gal group, n=12 (One way ANOVA and Tukey’ test).

Due to figure 1, diabetes was induced by D-gal in animals and associated to increased fasting blood glucose levels compared to all groups (p<0.001). Oral administration of myricitrin and vitamin E daily, the level of glucose was significantly decreased (p<0.001) compared to the D-gal group. In the D-gal group, insulin level increased compared to the control group (p<0.01). Thus, the administration of myricitrin and vitamin E caused a significant increase in insulin levels than control and D-gal groups (p<0.001). Myricitrin at doses 10 and 20 mg/kg had a similar impact of vitamin E on insulin secretion (Figure 2). The inappropriate changes in insulin biomarkers include HOMA-IR and HOMA-β observed in the D-gal group compared to control group as follows: 

A significant increase observed in HOMA-IR in the D-gal group compared to control group (p<0.001). Myricitrin at the lowest concentration (5 mg/kg) was reduced insulin resistance (p<0.05) compared to D-gal group. In contrast, vitamin E and myricitrin at higher concentrations did not improve this factor. Thus, the lowest dose of myricitrin is more effective than vitamin E (Figure 3). HOMA-β in the D-gal group had a significant decrease compared to control group (p<0.01). Treatment with myricitrin (p<0.001) and vitamin E (p<0.01) were increased this factor compared to D-gal group (Figure 4).

**Table 1:** The effect of myricitrin and vitamin E on lipid profile (mg/dl) and hepatic enzymes (U/L).

Factor/group	Control	D-gal	D-gal+5M	D-gal+10M	D-gal+20M	D-gal+VitE
Triglyceride	83.5±3.5	112±3^*^^*^	69.5±8.28^###^	57±2.08^*###^	59.5±1.5^*###^	87±7^#^
Cholesterol	82±4	96.4±1.4^*^^*^	76.25±7.82##	64±1.53###	75±8##	80.5±5.5#
LDL	17±4	27.5±1.5^*^^*^	15.5±3.96^#^	10.33±1.45^#^	15.9±3.10^#^	15.5±5.50^#^
VLDL	16.7±0.7	22.4±0.6^*^	13.87±1.67^###^	11.36±0.44^*###^	11.85±0.35^*###^	17.45±1.45^#^
HDL	46±3.00	46.5±0.5	47±2.61	42.33±1.85	47±5.00	47.5±1.50
ALP	111.2±8.9	139.1±4.2^**^	116.3±6.1##	107.1±2.1##	112.1±1.9##	107.2±1.1##
SGOT	379.5±21.5	481.1±5.1^*^	417.1±9.8	289.8±30.4###	418.1±8.1	383.1±2.9#
SGPT	69.5±0.5	93.5±4.5^*^	75.25±8.2	69.1±7.5#	85.1±0.1	89.5±3.5*

Data expressed as mean ± SEM. ^*^p<0.05, ^**^p<0.01 and, ^***^p<0.001 as compared with control group; ^#^p<0.05 and, ^###^p<0.001 as compared with D-gal group, n=12 (One way ANOVA and Tukey’ test).

**Table 2:** The effect of myricitrin and vitamin E on pancreas (diameter and number of Islets), and liver (RBCs accumulation and inflammation).

Factor/group		Control	D-gal	D-gal+5M	D-gal+10M	D-gal+20M	D-gal+VitE
Islet Diameter (µm)	175.3±13.4	53.3±5.4*	61.1±6.6*	150.2±11.6#	136.7±9.9*#	167.6±13.3#	
Islet Number (10^3^)	1.8±0.19	0.82±0.21*	1.78±0.31#	2.6±0.3*#	1.17±0.3*#	2/64±0.4*#	
RBCs accumulation	0.13±0.03	2.32± 0.36^***^	2.12±0.04^***^	0.21±0.05 ^#^^#^^#^	1.62±0.36^***^^#^	0.62± 0.26^*#^^#^	
Inflammation	0.12±0.02	2.13±0.31^***^	2.11±0.02^***^	0.65 ± 0.11 ^*#^^#^	1.93±0.25^***^	1.03±0.15^*#^	

Data expressed as mean ± SEM. ^*^p<0.05, ^**^p<0.01 and, ^***^p<0.001 as compared with control group; ^#^p<0.05 and, ^###^p<0.001 as compared with D-gal group, n=12 (One way ANOVA and Tukey’ test).

*The effect of myricitrin and vitamin E on lipid profile and hepatic enzymes.*

Due to the table 1, TG level significantly increased in the D-gal group compared to control group (p<0.01), which myricitrin (p<0.001) and vitamin E (p<0.05) can significantly reduce TG. 

Cholesterol increased in D-gal group compared to control group (p<0.01); thus, myricitrin (p<0.01) and vitamin E (p<0.05) were reduced cholesterol. LDL in D-gal group increased compared to control group (p<0.01) in which myricitrin and vitamin E were decreased LDL (p<0.05) compared to control group. 

HDL did not significantly change in all groups. D-gal resulted in a significant increase in the ALP level (p<0.01); however, myricitrin and vitamin E have improved this factor (p<0.01). SGOT level increased in D-gal group compared to control group (p<0.05). Myricitrin (p<0.001) and vitamin E (p<0.05) reduced this toxicity, myricitrin at 10 mg/kg was more effective than other doses. Vitamin E had a toxic impact on SGPT and increased this factor (p<0.05). Myricitrin at 10 mg/kg had a better effect on SGPT (Table 1).

**Figure 5:**
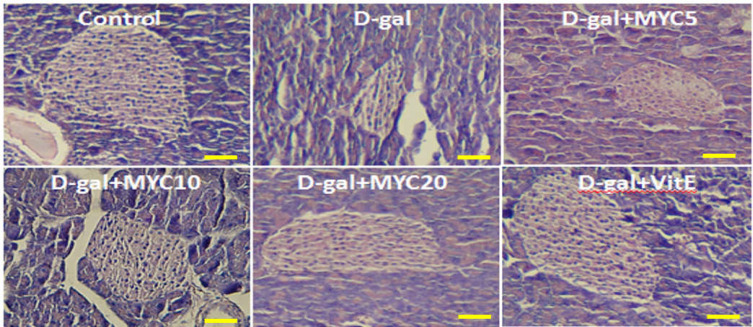
Effect of myricitrin and vitamin E on islets and pancreas histological analysis in D-gal diabetic mice. Scale bars: 50 µm

**Figure 6:**
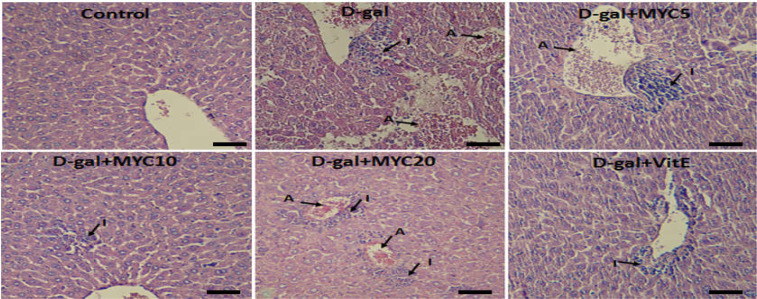
The effect of myricitrin and vitamin E on liver histological analysis in D-gal diabetic mice. I: inflammatory cells, A: accumulation of red blood cells, Scale bars: 50 µm

*Histopathological evaluation*

The diameter and numbers of the islets of Langerhans in the D-gal group significantly decreased compared with the control group (p<0.05). Vitamin E caused a significant increase in the diameter and numbers of the islets compared to D-gal group (p<0.05). Myricitrin at 10 mg/kg significantly increased the islet diameter and numbers, compared to D-gal group. Whereas Myricitrin at 20 mg/kg significantly decreased the islet diameter and numbers, compared to D-gal group. The exocrine part of pancreas was normal in different groups (Figure 5, Table 2). 

In the liver tissue, D-gal induced a significant increase in RBCs accumulation and inflammation (p<0.001). In D-gal+VitE group, RBCs accumulation and inflammation were significantly decreased compared to D-gal-treated animals (p<0.01 and p<0.05, respectively). Myricitrin at 10 mg/kg significantly decreased the histological criteria compared toD-gal group, whereas Myricitrin at the doses of 5 and 20 mg/kg could not significantly change the histological criteria of liver tissue (Figure 6, Table 2).

## Discussion

The present study indicated that glucose, insulin level, and HOMA-IR increased in D-gal-induced diabetic mice. Also, the beta-cell function was reduced. Along with the present results, previous studies are shown that D-gal induced diabetes and oxidative stress that played an essential role in the impairment of beta-cell function (17, 20). 

D-gal can be turned into superoxide anions (O2-), hydrogen peroxide (H2O2), and aldose are these alterations similarly associated to the natural aging process and exhibit a decrease in antioxidant enzyme level (21). Lipid metabolism and glucose impairments have been regarded as the main factors contributing to aging induced by D-gal in mice (22). Insulin resistance, pancreatic islet cell dysfunctions, and impaired glucose tolerance increase in the aging processassociated with diabetes (23, 24).

In previous studies, D-gal induced changes in liver enzymes and pancreas structure (6,17,20). Myricitrin and vitamin E were improved hyperglycemia and increased insulin level. At the lowest dose, myricitrin reduced insulin resistance, while vitamin E and myricitrin at higher concentrations did not develop this factor.Therefore, myricitrin at a lower dose was more effective than vitamin E administration on the improvement of hyperglycemia and insulin resistance. 

Flavonoids play a significant role in glucose uptake stimulation of peripheral tissue, insulin-mimetic action, and control the rate-limiting enzyme activity contributed to carbohydrate metabolism (25) and molecular cell pathway regulation, including improving pancreatic beta-cell proliferation and insulin secretion (9). The present results showed that treatment to the myricitrin improved D-gal-induced aging changes, including high blood glucose, hyperinsulinemia, and β-cell’s function marker. Thus, myricitrin administration elevated insulin plasma levels compared to D-gal group. Still, it can be proposed that this effect can exert during more ameliorated insulin secretion as a compensatory activity of β-cell, which is observable in islet diameter (as an elevated β-cell secretory index). Consistent with the findings of the present study, our previous investigation indicated that myricitrin can improve diabetes and complications induced by hyperglycemia in the in vivo and in vitro conditions in STZ-NA-induced type 2 diabetes in mice (26).

Reduced diameter and numbers of β-cells, combined with the increased RBCs accumulation and inflammation assumed to involve hyperglycemia and insulin resistance. Treatment with antioxidants could protect β-cells against oxidative stress as an effective treatment for diabetes (25). However, the present findings showed that myricitrin treatment increased the diameter and number of islets and reduced inflammation via improved hyperglycemia induced by apoptosis and pancreas toxicity.

Similar to our liver tissue results, higher plasma hepatic enzyme levels and histological disorders in mice injected with D-gal have been reported (6). In the present research, data indicate that myricitrin protects against hepatotoxicity associated to aging via improving metabolic disorders. However, treatment with vitamin E had a toxic effect on the SGPT level. Domitrovic et al. have been shown that myricitrin improved ALT, SGOT levels, and histopathological changes induced by D-gal in the liver through increasing glutathione level and cytochrome P450 expression, decreasing oxidative stress (13). Previous studies reported that treated mice with D-gal caused a significant increase in lipid profile (27). Myricitrin and vitamin E improved lipid profile changes resulted from D-gal in the mice.

The present findings indicated that myricitrin has a supporting impact on the liver and pancreas and metabolic disorders in aged mice induced by D- galactose. Thus, myricitrin indicated antidiabetic and hepatoprotective impacts on aged mice induced by D-gal.
